# Immunohistochemical Characterization of the Immune Response in Chronic Endometritis Caused by *Chlamydia trachomatis*

**DOI:** 10.3390/diagnostics16010164

**Published:** 2026-01-05

**Authors:** Ivett Miranda-Maldonado, Yareth Gopar-Cuevas, Salomón Álvarez-Cuevas, Guadalupe Gallegos-Avila, Jesús Ancer-Rodríguez, Marta Ortega-Martínez, Gilberto Jaramillo-Rangel

**Affiliations:** Department of Pathology, School of Medicine, Autonomous University of Nuevo León, Monterrey 64460, Mexico; ivettmiranda77@gmail.com (I.M.-M.); yareth.goparcu@uanl.edu.mx (Y.G.-C.); salomon.alvarezcu@gmail.com (S.Á.-C.); ggallegos.avila8@gmail.com (G.G.-A.); ancerrodriguezj@gmail.com (J.A.-R.); marta.ortegamrt@uanl.edu.mx (M.O.-M.)

**Keywords:** *Chlamydia trachomatis*, immune cells, chronic endometritis, miscarriage

## Abstract

In 2020, 128.5 million new chlamydia infections were reported worldwide in adults aged 15–49 years. Notably, the prevalence of *Chlamydia trachomatis* infection in pregnant women varies between 2% and 35%, correlating with increased risks of low birth weight, preterm birth, and neonatal death. *C. trachomatis* is a leading preventable cause of miscarriage. Recurrent first-trimester pregnancy loss can be induced by asymptomatic chlamydia infection through the immune response. In this study, we performed immunohistochemical characterization of the immune response in endometrial tissue biopsies from women diagnosed with chronic endometritis caused by *C. trachomatis*. Hematoxylin and eosin staining was used for histological evaluation of endometrial biopsies, and immunohistochemical detection was performed for the following markers: CD138, CD45, CD3, CD4, CD8, CD20, CD56, and CD68. As a result, we observed the presence of edematous tissue with hemorrhage; we also observed a heightened inflammatory response with the presence of plasma cells, CD4 and CD8 T lymphocytes, B lymphocytes, NK cells, and macrophages. The findings described here can help better understand the disease and its histopathological diagnosis.

**Figure 1:**
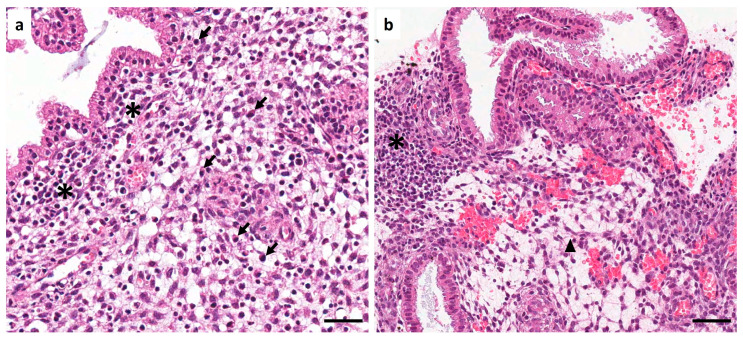
Chronic endometritis refers to persistent inflammation of the endometrium, often presenting asymptomatically [1]. This disease is frequently associated with microbial infections, particularly those caused by *Ureaplasma urealyticum*, *Mycoplasma hominis*, and *Chlamydia trachomatis* [2]. Colonization of the endometrium by these bacteria has been associated with decreased implantation rates and increased miscarriage rates. Bacterial endotoxins induce a heightened immune response, stimulating the production of pro-inflammatory cytokines that create an environment that can damage the embryo [3,4]. In this study, immunohistochemical characterization of the immune response was performed on endometrial biopsies from women diagnosed with chronic endometritis caused by *C. trachomatis*. Thirty patients suffering from infertility and/or chronic genitourinary infection refractory to treatment were selected. The age of the patients ranged from 22 to 49 years, with a median of 33 years. All patients included in the study had confirmed *C. trachomatis* infection in uterine secretions and endocervical brushing cytology, as determined by PCR and the Chlamydia direct IF identification kit (BioMérieux, SA. Lyon, France). Patients receiving treatment with steroidal and nonsteroidal anti-inflammatory drugs were not included in the study. Endometrial biopsies were embedded in paraffin according to standard histopathological procedures. Five-µm-thick sections were staining with H&E according to the standard technique and immunostained with a standard protocol previously used in our laboratory [5]. The monoclonal antibodies used were CD138, CD20, CD56, CD68 (Biocare Medical, Pacheco, CA, USA, dilution 1:100), CD45, CD3, CD4, and CD8 (DAKO, Glostrup, Denmark, prediluted). All markers analyzed were positive in all patients. The immunohistochemical images presented are representative of all cases examined. (**a**) First, hematoxylin and eosin (H&E)-stained endometrial biopsies were analyzed. The presence of lymphocytes (asterisk) and plasma cells (arrows) in the endometrial stroma was observed; (**b**) furthermore, a large area of edematous tissue with hemorrhage (arrowhead) with a lymphocytic inflammatory infiltrate (asterisk) in the endometrial stroma was observed. Scale bar 20 µm.

**Figure 2:**
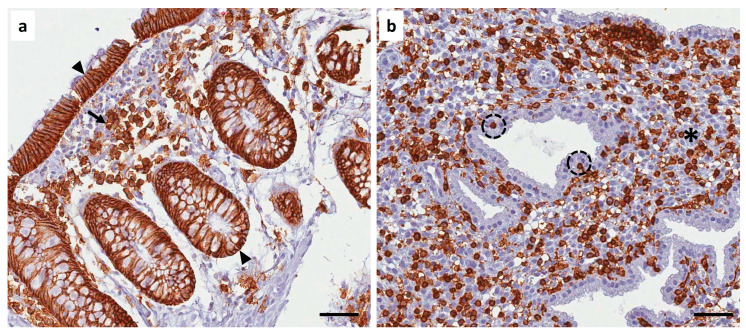
The gold standard for diagnosing chronic endometritis is the identification of plasma cells in endometrial biopsies through histopathological analysis [3]. However, the accuracy of the diagnosis may be compromised because the detection of plasma cells with H&E stain is difficult as the endometrium may have mononuclear cell infiltration, stromal cell proliferation, the presence of plasmacytoid-appearing stromal cells, or a prominent inflammatory reaction of the endometrium in a late secretory phase [6]. Therefore, histopathologic evaluation using immunohistochemistry for plasma cells marker CD138 (syndecan-1) is the most reliable diagnostic method for chronic endometritis [7]. It is essential to consider that, despite the usefulness of CD138 immunostaining for diagnosing chronic endometritis, caution should be exercised when interpreting the results, as endometrial epithelial cells constitutively express CD138 [8]. (**a**) Detection of plasma cells by immunohistochemistry with the CD138 marker. Plasma cells were present in the endometrial stroma (arrows); furthermore, a positive signal for CD138 was seen in the lining and glandular epithelial cells (arrowheads) [9]. On the other hand, leukocytes represent between 10 and 20% of normal endometrial stromal cells. An increase in the percentage of leukocytes indicates the development of an inflammatory process in the endometrium [10]. (**b**) Therefore, we performed leukocyte detection by immunohistochemistry using the CD45 marker. A heightened inflammatory response was observed, with abundant leukocytes present in the endometrial stroma (asterisk) and the glandular epithelium (dotted circle). Scale bar 20 µm.

**Figure 3:**
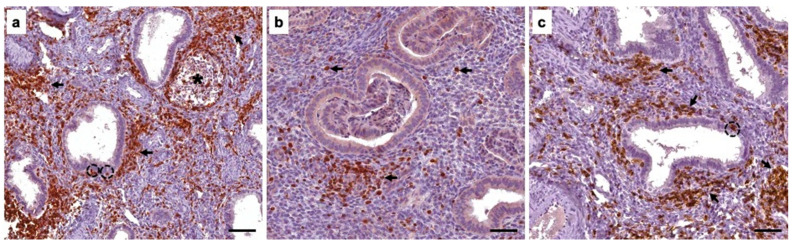
The leukocyte profile of the normal endometrial stroma is primarily composed of lymphocytes, with T lymphocytes constituting the predominant population, because they comprise 40–60% of the leukocytes present in the endometrium [11]. (**a**) We analyzed T lymphocytes in the endometrium of patients by immunohistochemistry for the CD3 marker. Abundant T lymphocytes were observed in the endometrial stroma (arrows) and in the area surrounding the lymphoid follicles (asterisk); interestingly, T lymphocytes were also present in the glandular epithelium (dotted circles). We also analyzed the CD4 and CD8 markers by immunohistochemistry to determine the presence of helper and cytotoxic T lymphocytes, respectively. The presence of moderate helper (**b**) and abundant cytotoxic (**c**) T lymphocytes was observed in the endometrial stroma (arrows). It is important to note that the presence of cytotoxic T lymphocytes was also observed in the glandular epithelium of the endometrium (dotted circle). Scale bar 20 µm.

**Figure 4:**
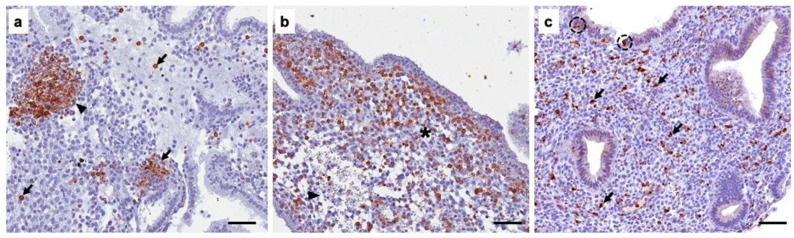
B lymphocytes comprise <1% of the total lymphocyte population of the endometrial stroma [12]. (**a**) We evaluated the B lymphocyte population by immunohistochemistry for the CD20 marker. Abundant B lymphocytes were observed in the endometrial stroma, forming lymphoid follicles (arrowhead). B lymphocytes were also observed outside the lymphoid follicles in the endometrial stroma (arrows). On the other hand, under normal conditions, the percentage of natural killer (NK) cells found in the endometrium ranges from 0.9% to 5.3% [13]. (**b**) NK cells were evaluated by immunohistochemistry for the CD56 marker. Abundant NK lymphocytes were observed in the endometrial stroma (asterisk) and surrounding a large area of edematous tissue (arrowhead). As for macrophages, they comprise approximately 10% of the total leukocytes present in the endometrium [14]. (**c**) We evaluated the presence of macrophages in the endometrium by detecting CD68 expression using immunohistochemistry. We observed the presence of abundant macrophages in the endometrial stroma (arrows), as well as in the lining epithelium (dotted circles). Scale bar 20 µm. In this work, we characterize the immune response in chronic endometritis caused by *C. trachomatis*. Unlike other related articles, this work presents more immunohistochemical images and/or evaluates more markers of specific leukocyte populations [9,15]. Furthermore, the images reveal understudied morphological findings, such as the presence of certain leukocyte groups in the glandular and lining epithelium (Figure 3a,c and Figure 4c). Further research is needed to understand the significance of these findings.

## Data Availability

The data presented in this study are available on request from the corresponding author due to ethical restrictions.

## Abbreviations

The following abbreviations are used in this manuscript:

µm	Micrometers
H&E	Hematoxylin and eosin
IF	Immunofluorescence
PCR	Polymerase chain reaction
